# Phylogenetic sleuthing reveals pair of paralogous genes

**DOI:** 10.7554/eLife.17224

**Published:** 2016-05-31

**Authors:** Jamie E Henzy, Welkin E Johnson

**Affiliations:** Biology Department, Boston College, Chestnut Hill, United States; Biology Department, Boston College, Chestnut Hill, United Stateswelkin.johnson@bc.edu

**Keywords:** innate immunity, mRNA modification, translation, gene conversion, phylogeny, host-virus interactions, Human, Mouse, *S. cerevisiae*, Virus

## Abstract

The complex evolutionary history of the *IFIT* family of antiviral genes has been shaped by continuous interactions between mammalian hosts and their many viruses.

**Related research article** Daugherty M, Schaller A, Geballe A, Malik H. 2016. Evolution-guided analyses reveal diverse antiviral specificities encoded by IFIT1 genes in mammals. *eLife* **5**:e14228. doi: 10.7554/eLife.14228

**Image** The yeast assay developed by Daugherty et al.

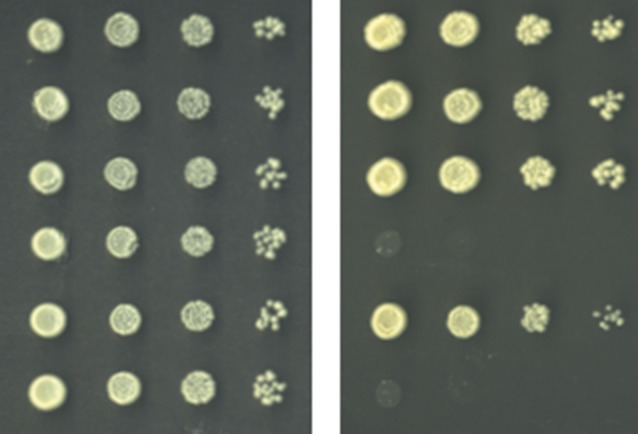

The genes involved in innate immunity face special challenges that other genes do not. For example, in order to target invading viruses, the immune system needs to distinguish between viral ("non-self") components and "self" components that are meant to be in the host. Complicating this task is the wide assortment of potential pathogens that the host may encounter, combined with the ability of most pathogens to readily evolve to escape the host's immune system. This "moving target" requires evolutionary innovation on the part of the host, which is a tall order for vertebrates because they evolve sluggishly.

Now, in eLife, Matthew Daugherty of the Fred Hutchinson Cancer Research Center (FHCRC) and co-workers report how they have used a combination of phylogenetic sleuthing and a novel assay in yeast to explore the evolution of a family of antiviral proteins called IFIT proteins (Daugherty et al., 2016). These proteins are components of the innate immune system in mammals (Fensterl and Sen, 2015).

When a virus is detected, a signaling protein called interferon is released and it induces an antiviral state by upregulating a whole slew of interferon-stimulated genes. *IFIT* genes are among the most highly upregulated of these genes, and IFIT proteins target a wide range of viruses (Diamond and Farzan, 2013). It is known that *IFIT* genes are encoded by a complex multigene locus that varies among different mammalian lineages: for example, a common mouse has six intact copies of *IFIT*-like genes, whereas a rat has four (Liu et al., 2013). However, despite their importance to innate immunity, there is much we do not know about IFIT proteins.

One puzzle involves the ability of an IFIT protein previously known as IFIT1 to distinguish between cellular (mammalian) mRNAs and viral mRNAs. Mammalian mRNA advertises its selfness by adding a methyl group to the first ribose sugar of the mRNA molecule to produce a "cap1" structure; the unmethylated mRNA molecule is said to have a "cap0" structure (Figure 1; Banerjee, 1980). However, many viruses have evolved ways to fly under the radar either by "snatching" cap1 structures from host mRNAs, or by encoding a methyltransferase enzyme that allows them to make their own cap1 structures (Hyde and Diamond, 2015).

**Figure 1.**
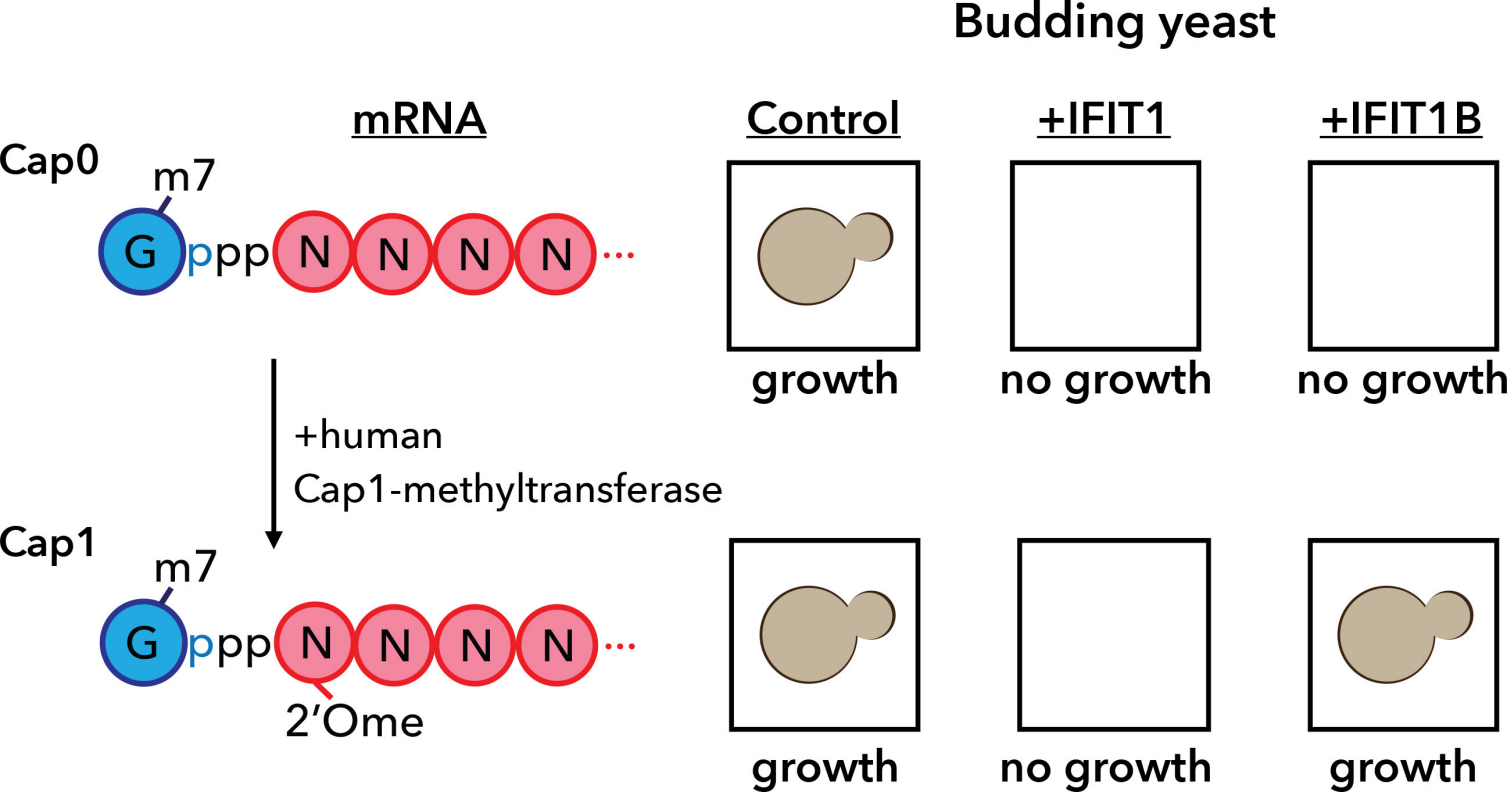
A yeast assay to discriminate between the antiviral proteins IFIT1 and IFIT1B. IFIT1 and IFIT1B both inhibit the growth of budding yeast (*Saccharomyces cerevisiae;* top row, middle and right). Co-expressing human Cap1-methyltransferase (which converts cap0-mRNA to cap1-mRNA by adding a methyl group to the first ribose sugar; left) rescued yeast cells expressing IFIT1B (bottom row, right), but not yeast cells expressing IFIT1B (bottom row, middle). Daugherty et al. used this assay to study a wide range of IFIT1/IFIT1B homologs. IFIT: interferon-induced tetratricopeptide repeats.

This disguise appears to work against mouse IFIT1 protein, which exclusively targets cap0 mRNAs, leaving cap1 mRNAs unscathed (Diamond, 2014). Human IFIT1 protein, by contrast, is not tricked by the disguise and targets both cap0 and cap1 viral mRNAs. This suggests that human IFIT1 protein recognizes viral mRNA by a different and as-yet-unknown means. A simple explanation for this difference could be that functional specialization, perhaps driven by challenges from different pathogens, drove the *IFIT1* gene to diverge in the mouse and human lineages. This would make the *IFIT1* genes orthologs.

Daugherty and co-workers – Aaron Schaller and Harmit Malik, both from FHCRC, and Adam Geballe from FHCRC and the University of Washington School of Medicine – examined this assumption by comparing the *IFIT* locus among a wide panel of mammalian genomes. This analysis pointed to a gene duplication event that occurred at the *IFIT1* locus early in mammalian evolution, more than 100 million years ago. This means that mouse *IFIT1* and human *IFIT1* are paralogs (caused by a gene duplication event) rather than orthologs (caused by a speciation event), so the authors renamed the mouse gene *IFIT1B*.

Armed with this new insight into the relationship between human IFIT1 and mouse IFIT1B, Daugherty et al. demonstrated that these two paralogs indeed have distinct functions. To do this, they made use of the fact that budding yeast produces only cap0 structures (Banerjee, 1980). When they expressed mouse IFIT1B in yeast, it targeted cap0 mRNA, in turn inhibiting growth of the yeast, as expected. Growth was rescued when human cap1-methyltransferase was co-expressed with IFIT1B (Figure 1). Human IFIT1, on the other hand, behaved differently. Like mouse IFIT1B, it also inhibited cap0 mRNA and inhibited the growth of the yeast: however, growth was not rescued by co-expressing cap1-methyltransferase. This suggests that IFIT1 depends on something other than cap status to find its target mRNAs. Interestingly, orthologs of IFIT1B from two primates – gibbons and African green monkeys – exhibited the same cap0-targeting behavior as mouse IFIT1B, despite rodents and primates having diverged more than 100 million years ago. Human IFIT1B, by contrast, has lost this particular function.

This study illustrates how a family of vertebrate genes involved in pathogen resistance is continually shaped by the dynamic nature of the multigene locus in which it resides. Such loci frequently undergo duplication, recombination, and gene conversion events (Eirín-López et al., 2012; Nei et al., 1997) that can significantly alter the immune repertoire of various species. Gene duplication events, in particular, can result in relaxed selective pressure on one of the copies, allowing the acquisition of novel functions (Lynch and Conery, 2000; Ohno, 1970). As shown here, such an event is the likely basis for the different functions of IFIT1 and IFIT1B. Indeed, based on this study, these two genes appear to have been the most active of the mammalian *IFIT* genes, undergoing frequent gene birth and loss, as well as recurrent homogenization.

A number of questions remain unanswered: for instance, how does IFIT1 recognize viral mRNA? And what is the function of human IFIT1B (if, indeed, it has a function)? However, the insights that Daugherty et al. glean from applying phylogenetic analyses to functional studies elegantly underscore the value of multigene loci as facilitators of adaptive responses in the ongoing interplay between vertebrates and their pathogens.
